# IEX-1 deficiency induces browning of white adipose tissue and resists diet-induced obesity

**DOI:** 10.1038/srep24135

**Published:** 2016-04-11

**Authors:** Mohd Shahid, Ammar A. Javed, David Chandra, Haley E. Ramsey, Dilip Shah, Mohammed F. Khan, Liping Zhao, Mei X. Wu

**Affiliations:** 1 The Wellman Center for Photomedicine, Massachusetts General Hospital (MGH) and Department of Dermatology, Harvard Medical School (HMS), Boston, Massachusetts 02114 USA; 2 Department of Anesthesia, Critical Care and Pain Medicine, Shriners Hospitals for Children, MGH and HMS, Boston, MA 02114, USA; 3 Department of Molecular Biology, MGH and HMS, Boston, Massachusetts USA; 4 Harvard-Massachusetts Institute of Technology Division of Health Sciences and Technology, Boston, Massachusetts USA

## Abstract

Chronic inflammation plays a crucial role in the pathogenesis of obesity and insulin resistance. However, the primary mediators that affect energy homeostasis remain ill defined. Here, we report an unexpected role for immediate early response gene X-1 (IEX-1), a downstream target of NF-κB, in energy metabolism. We found that IEX-1 expression was highly induced in white adipose tissue (WAT) in both epidydmal and subcutaneous depots but not in interscapular brown adipose tissue (BAT) in mice fed a high fat diet (HFD). Null mutation of IEX-1 protected mice against HFD-induced adipose and hepatic inflammation, hepatic steatosis, and insulin resistance. Unexpectedly, IEX-1 knockout (IEX-1^−/−^) mice gained markedly less weight on HFD for 20 weeks as compared to wild-type (WT) littermates (37 ± 3 versus 48 ± 2 gm) due to increased energy expenditure. Mechanistically, we showed that IEX-1 deficiency induced browning and activated thermogenic genes program in WAT but not in BAT by promoting alternative activation of adipose macrophages. Consequently, IEX-1^−/−^ mice exhibited enhanced thermogenesis (24 ± 0.1 versus 22 ± 0.1 kcal/hour/kg in WT mice) explaining increased energy expenditure and lean phenotype in these mice. In conclusion, the present study suggests that IEX-1 is a novel physiological regulator of energy homeostasis via its action in WAT.

Obesity is one of today’s most alarming public health problems because of its high prevalence (59 million Americans) and its association with a wide range of chronic diseases, such as type 2 diabetes, atherosclerosis, hypertension, non-alcoholic fatty liver, immune-mediated disorders, and some types of cancers^1^. It is associated with chronic low-grade active inflammation in important metabolic tissues including adipose tissue and liver. The chronic inflammation alters glucose and lipid metabolism and results in excessive energy storage and insulin resistance^2^,^3^,^4^,^5^,^6^. Recent studies have provided crucial evidence that an innate immune response and subsequent inflammation occurs at a much earlier stage than the inception of obesity and critically contributes in its pathogenesis^2^,^4^,^5^,^6^. Specifically, NF-κB, a central inflammatory mediator, plays a major role in the diet-induced inflammation. Blockade of NF-κB and its downstream mediators not only protects mice against diet-induced insulin resistance but also from obesity^5^,^7^,^8^, suggesting a crucial nexus between inflammation and energy expenditure. Despite the strong evidence of involvement of inflammation in metabolism imbalance, the primary mediators that impair energy balance during high fat intake are not fully defined.

Immediate early response gene X-1 or immediate early response 3 (IEX-1 or IER3) is an early stress inducible gene that is a direct downstream transcriptional target of NF-κB. Inhibiting IEX-1 blocks several functions of NF-κB^9^,^10^,^11^,^12^. IEX-1 is highly expressed in macrophages that are responsible for majority of the inflammation associated with obesity in humans and mice^11^,^13^,^14^. Its expression increases in macrophages and vasculature in mice fed with a high fat diet (HFD)^15^. We have previously reported that macrophages lacking IEX-1 produced only a diminished inflammatory response to *Leishmania major* infection^14^ or dextran sodium salt (DSS)-induced colitis^16^ in mice, emphasizing an important role IEX-1 in inflammation. Based on these observations, we hypothesized that IEX-1 may be required for HFD-induced inflammation and contributes to development of insulin resistance. Here, we report an unexpected requirement for IEX-1 in HFD-induced obesity and inflammation. HFD feeding in mice induced IEX-1 expression in white adipose tissue (WAT) both in epidydmal and subcutaneous inguinal depots. Mice lacking functional IEX-1 were not only protected from HFD-induced inflammation and insulin resistance but also from obesity. Mechanistically, IEX-1 deficiency induced browning and increased thermogenic genes expression in epidydmal and subcutaneous WAT by sustaining alternatively activated macrophages (AAMs) in WAT, without altering brown adipose tissue (BAT) function. The browning of WAT in turn enhanced thermogenesis and thereby increased energy expenditure in IEX-1 knockout (IEX-1^−/−^) mice on HFD, providing a mechanism whereby IEX-1 deficiency inhibits obesity development. Thus, IEX-1 represents a novel candidate protein involved in physiological regulation of energy homeostasis and may play an important role in the pathogenesis of the metabolic disorders.

## Results

### IEX-1 expression increases in white adipose tissue after HFD feeding

**Figure 1:**
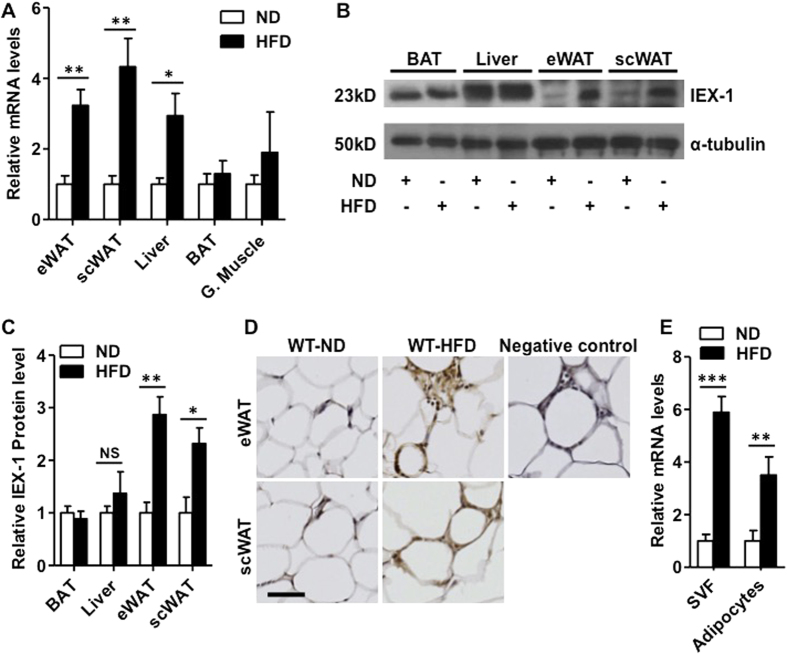
HFD feeding induces IEX-1 expression in white adipose tissue. (**A**) qRT-PCR analysis for IEX-1 mRNA levels in tissues obtained from wild-type (WT) mice fed with high fat diet (HFD) or normal diet (ND) for 8 weeks, demonstrating that HFD induced IEX-1 gene expression in epidydmal (eWAT), subcutaneous WAT (scWAT), and liver of mice (*n* = 4). (**B**) Immunoblotting of the whole cell lysate of indicated tissues obtained from the mice described in (**A**) confirmed increased IEX-1 expression in eWAT and scWAT but not in liver or brown adipose tissue (BAT) in response to HFD feeding. α-tubulin was used as a loading control for IEX-1. (**C**) Quantitative analysis of immunoblots shown in (**B**). (**D**) Immunohistochemistry staining of eWAT and scWAT sections of these mice shows increased IEX-1 signal (brown color) in HFD-fed mice as compared to those fed with ND. Note the enhanced IEX-1 signal in infiltrated immune cells in crown like structure both in eWAT and scWAT. No primary antibody was added in negative control sections. (**E**) qRT-PCR analysis for IEX-1 mRNA levels in stromal vascular fraction (SVF) and adipocytes of eWAT obtained from mice fed with HFD or ND for 8 weeks, indicating a robust increase in IEX-1 mRNA levels in SVF after HFD feeding (*n* = 4). Shown in (**B**,**D**) are representative images of 3 independent experiments. *p < 0.05, **p < 0.01, ***p < 0.001 as indicated, G. Muscle - Gastrocnemius muscle, NS – non significant. Bar in D = 50 μM.

To investigate a role of IEX-1 in HFD-induced obesity we analyzed IEX-1 expression in different metabolic organs of wild-type (WT) mice fed with ND (normal diet) or HFD for 8–10 weeks. As shown in Fig. 1A, HFD induced significant increases in the transcript levels of IEX-1 in epidydmal WAT (eWAT), subcutaneous WAT (scWAT), and liver by 3.2 ± 0.4, 4.3 ± 0.8, and 2.4 ± 0.4-fold, respectively as compared to ND-fed mice (Fig. 1A). No significant changes in IEX-1 gene expression were observed in brown adipose tissue (BAT) and gastrocnemius muscle of HFD-fed mice. The changes in mRNA level were also reflected in IEX-1 protein levels in eWAT, scWAT, but not in liver of these mice (Fig. 1B,C). Immunohistological analysis further demonstrated an increased IEX-1 activity in the sections of formalin-fixed eWAT and scWAT (Fig. 1D). IEX-1 signal in eWAT and scWAT sections was particularly strong in infiltrated immune cells in the crown like structures, suggesting that HFD feeding induced IEX-1 expression in infiltrated immune cells that may have contributed to increased IEX-1 expression observed in WAT. To confirm this we separated stromal vascular fraction (SVF) from adipocyte in eWAT. HFD induced a 6-fold increase in IEX-1 expression in SVF and 3-fold increase in adipocytes (Fig. 1E). These data demonstrate that HFD induces IEX-1 expression predominantly in WAT that is attributed at least in part to the increased IEX-1 expression in the infiltrated immune cells.

### IEX-1 deficiency renders mice refractory to HFD-induced obesity

**Figure 2:**
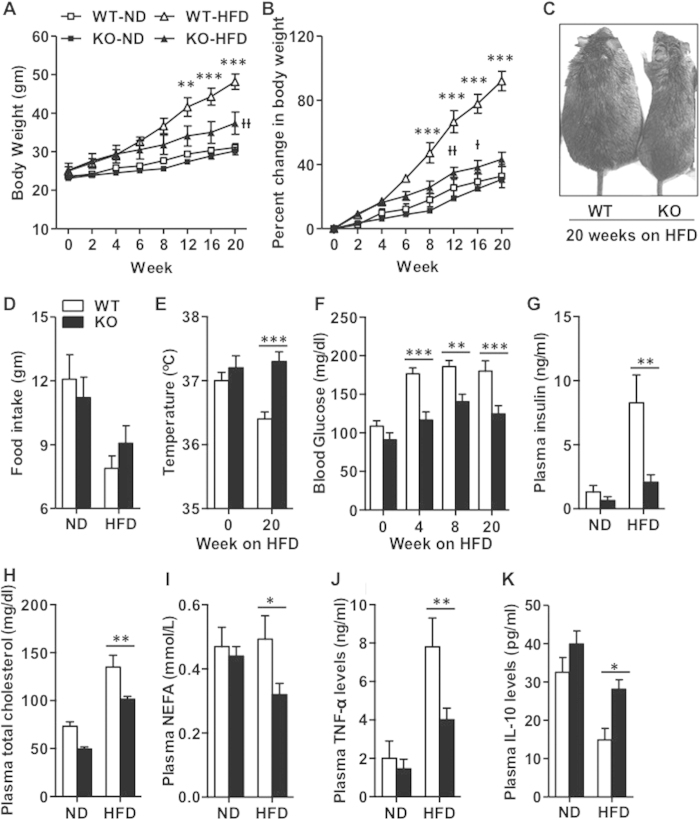
Null mutation of IEX-1 resists the development of HFD-induced obesity and hyperinsulinemia. (**A**) The absolute and (**B**) percent change in body weight of IEX-1^−/−^ (KO) and their WT littermates fed with HFD or ND over 20 weeks, illustrating installation of obesity in WT but not in IEX-1^−/−^ mice (*n* = 7). (**C**) Shown in inset one KO and WT mouse each representing respective group of mice fed with HFD for 20 weeks. Measurements of (**D**) total daily food intake, (**E**) rectal temperature, and fasted (**F**) blood glucose, and (**G**) plasma insulin in KO (fill) and WT (empty) mice, demonstrate increased food consumption and rectal temperature but reduced glucose and insulin levels in KO mice on HFD as compared to WT control *(n* = 4–7). Fasted plasma (**H**) cholesterol and (**I**) non-esterified fatty acids (NEFA) are also lower in KO than WT mice. ELISA assay in plasma obtained from mice described in (**A**) revealed lower levels of circulating TNF-α whereas higher levels of IL-10 in KO than in WT mice after HFD feeding for 20 weeks (*n* = 4–6). *p < 0.05, **p < 0.01, ***p < 0.001 WT-HFD vs KO-HFD; and ^†^p < 0.05, ^††^p < 0.01 KO-HFD vs KO-ND in (**A,B**) or as indicated.

To investigate a physiological importance of IEX-1 in obesity, we fed IEX-1^−/−^ mice^17^ and their WT littermate with either a HFD or ND for 20 weeks. IEX-1^−/−^ mice grow and breed normally and weigh similar to WT mice when fed ND^17^. Likewise, body weight was similar between WT and IEX-1^−/−^ mice on ND over the entire study period (Fig. 2A). In contrast, after 20 weeks of HFD consumption WT mice gained 92% in their body weight (48 ± 2 gm from 25 ± 1), whereas, IEX-1^−/−^ mice had their body weight increased by only 44% (37 ± 3 gm from 25 ± 2; Fig. 2A,B), demonstrating that IEX-1^−/−^ mice were resistant to HFD-induced obesity (Fig. 2C). Paradoxically, IEX-1^−/−^ mice appeared to consume more food than WT littermates when mice were fed with HFD, although it did not reach statistical significance (Fig. 2D). IEX-1^−/−^ mice also maintained greater core body temperature than WT mice on HFD (37.3 ± 0.1 vs 36.4 ± 0.1 °C; Fig. 2E), suggestive of increased thermogenesis in this strain of mice only when given HFD. This observation is consistent with normal body weight of IEX-1^−/−^ mice when on ND.

We went on to investigate the effect of IEX-1 deficiency on glucose and lipid homeostasis. There was no significant difference in fasting blood glucose and plasma insulin levels between WT and IEX-1^−/−^ mice on ND in agreement with no alteration in food intake and body weight on ND. HFD robustly increased fasting blood glucose (Fig. 2F) and plasma insulin levels (Fig. 2G) in WT mice, an effect that was markedly inhibited in IEX-1^−/−^ mice. HFD feeding also raised plasma total cholesterol (Fig. 2H) but not non-esterified fatty acid (NEFA; Fig. 2I) in WT mice; however, the levels were once again significantly lower in IEX-1^−/−^ mice. Furthermore, HFD markedly elevated plasma levels of inflammatory cytokines TNF-α (Fig. 2J) and decreased the levels of anti-inflammatory cytokine IL-10 (Fig. 2K). These effects were inhibited in IEX-1^−/−^ mice, giving rise to higher level of IL-10 in the absence than in presence of IEX-1. Taken together, these data suggest that IEX-1 deficiency protects mice against HFD-induced weight gain, impairment in glucose and lipid metabolism, and systemic inflammation.

### IEX-1 deficiency improves glucose metabolism

**Figure 3:**
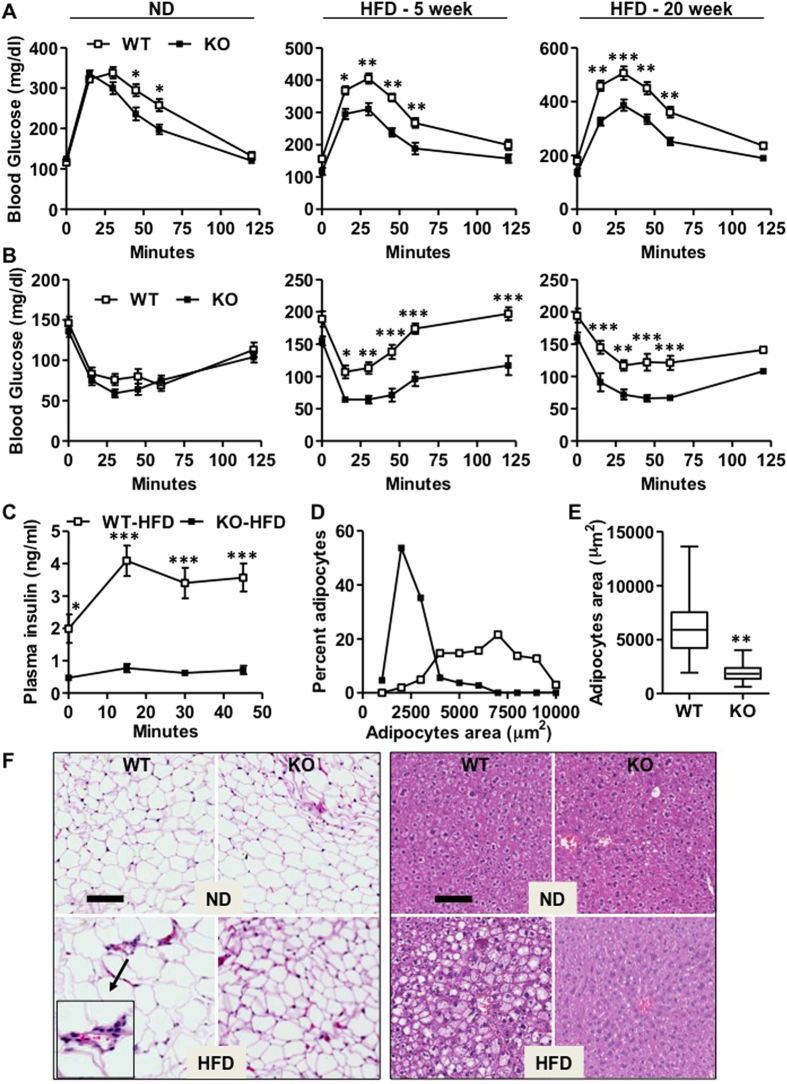
Increased glucose clearance, reduced adipocytes enlargement and hepatic steatosis in IEX-1 deficiency. (**A**) Intraperitoneal glucose tolerance (IGTT) and (**B**) insulin tolerance (ITT) tests demonstrate that HFD feeding for 5 or 20 weeks impaired glucose clearance and installed insulin resistance in WT mice (empty). These effects were markedly inhibited in IEX-1 KO mice (fill), indicating improved glucose metabolism in IEX-1 deficiency (*n* = 7). (**C**) ELISA assay for plasma insulin concentration shows a robust increase in circulating insulin levels in WT mice on HFD in response to glucose administration during IGTT, an effect that was abrogated in KO mice (*n* = 4). Quantification of adipocytes diameter on eWAT sections shown in (**F**) indicated (**D**,**E**) diameter distribution shifting toward smaller sizes in KO compared with WT mice. (**F**) Histological analysis by H&E staining of eWAT (left panel) and liver (right panel) displayed increase in adipocytes area and liver fat accumulation (hepatic steatosis) in WT mice after 20 weeks on HFD. These effects were markedly inhibited in IEX-1 KO mice. Shown in (**F**) is one representative result each of 3 independent experiments. *p < 0.05, **p < 0.01, ***p < 0.001 versus corresponding KO value or as indicated. Bars in F = 100 μM.

To determine the metabolic status of mice in the absence of IEX-1, we performed intraperitoneal glucose tolerance test (IGTT) and insulin tolerance test (ITT) after 20 weeks of HFD or ND feeding. IEX-1^−/−^ mice displayed significantly faster glucose clearance than their WT counterparts during both IGTT (right panel of Fig. 3A) and ITT (right panel of Fig. 3B). No major difference was observed between two strains of mice when fed with ND except that KO mice displayed a modest increase in glucose clearance during IGTT (right panels of Fig. 3A,B). Moreover, glucose administration during IGTT induced a robust increase in plasma insulin levels in WT mice but produced only a modest effect in IEX-1^−/−^ mice, suggesting improved insulin sensitivity in latter (Fig. 3C). Likewise, after 5 weeks of short period of HFD consumption, when there was only a marginal difference in the body weight between two strains of mice, WT mice already showed signs of impaired glucose metabolism and insulin resistance which was not the case for IEX-1^−/−^ mice (mid panels of Fig. 3A,B). These data suggest that improved glucose metabolism in IEX-1 deficiency may not be entirely a consequence of reduced weight gain in KO mice after HFD. Furthermore, chronic HFD feeding in WT mice caused enlargement of adipocytes, infiltration of immune cells, and fatty liver (steatosis) as reported previously (Fig. 3D–F)^5^,^18^. These effects of HFD feeding were however markedly attenuated in IEX-1^−/−^ mice even after 20 weeks (Fig. 3D–F). Adipocyte diameter distribution shifted toward smaller size in IEX-1 deficiency (Fig. 3D,E). These data suggest that IEX-1 deficiency improves HFD-induced insulin resistance and hepatic steatosis that cannot be explained by the reduced weight gain in KO mice after HFD.

### IEX-1 deficiency inhibits HFD-induced adipose and hepatic inflammation

**Figure 4:**
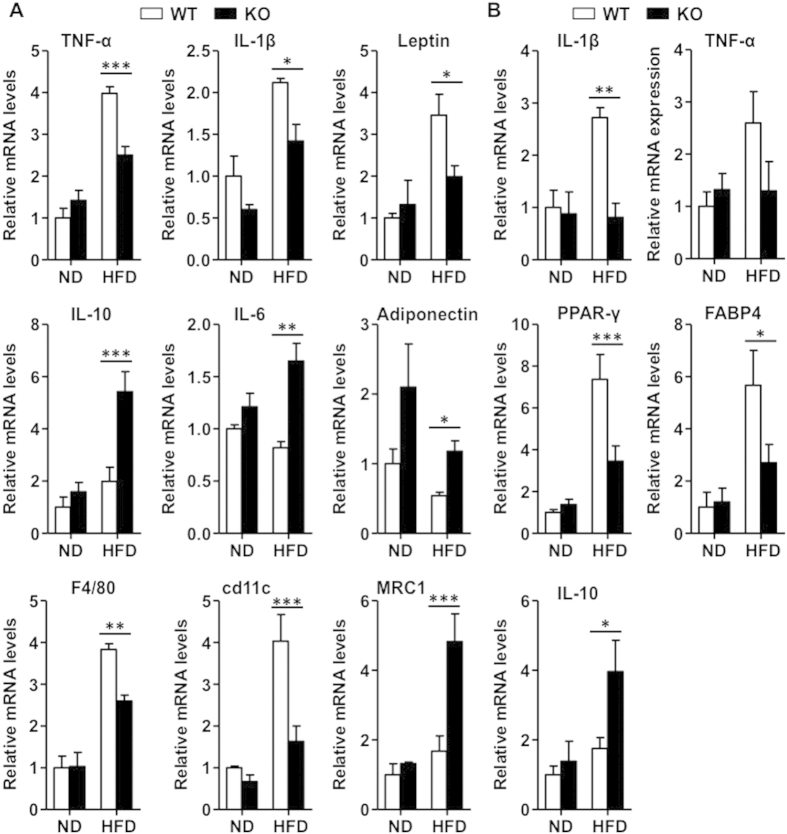
IEX-1 KO mice are protected from HFD-induced adipose and hepatic inflammation. (**A**) The qRT-PCR analysis for inflammatory cytokines genes demonstrated that HFD feeding for 8 weeks increased the expression of TNF-α, IL-1β, and leptin concomitant with a decrease in adiponectin levels in eWAT of WT mice (empty). These effects were inhibited in IEX-1 KO mice (fill). HFD did not alter the expression of (mid panels (**A**)) IL-10 and IL-6 in eWAT of WT mice, however, the levels were higher in KO mice after 8 weeks of HFD. HFD also induced the expression of (bottom panels (**A**)) F4/80 and cd11c, the markers for total and classically activated macrophages (CAMs), respectively, in eWAT, effects that were either partially inhibited or absent in KO mice. The expression of MRC1, a marker of alternatively activated macrophages (AAMs), did not change significantly with HFD feeding, however, its levels were highly induced in KO mice. (**B**) HFD feeding also increased the expression of (top panels (**B**)) IL-1β and TNF-α and (mid panels (**B**)) adipogenesis genes PPAR-γ and FABP4 in liver, effects that were abrogated in KO mice. Hepatic (bottom panels (**B**)) IL-10 expression did not change in WT mice with HFD feeding, however, its levels were increased in KO mice (*n* = 4–6). *p < 0.05, **p < 0.01, ***p < 0.001 as indicated.

HFD induces chronic inflammation in metabolic tissues that impairs insulin signaling leading to insulin resistance^7^,^19^. Since IEX-1^−/−^ mice sustained insulin sensitivity even after 20 weeks of HFD, we examined the inflammatory status of adipose tissue and liver that are involved in insulin signaling in mice fed with HFD. As expected, WT mice fed with HFD for 8 weeks showed robust increases in the expression of pro-inflammatory cytokines TNF-α, IL-1β and leptin, with a concomitant decrease in anti-inflammatory cytokine adiponectin in eWAT (Fig. 4A). These effects were markedly diminished in IEX-1^−/−^ mice. On the contrary, IL-6 and anti-inflammatory cytokine IL-10 expression did not alter after HFD in WT mice, however, the levels of these cytokines were elevated in KO mice (mid-panels of Fig. 4A). Similarly, the expression levels of F4/80, a marker of macrophage, and cd11c, a marker of classically activated macrophages (CAMs) were highly increased in eWAT of WT mice on HFD feeding, indicative of increased infiltration of pro-inflammatory macrophage. However, these effects were inhibited in IEX-1^−/−^ mice (bottom panels of Fig. 4A). In contrast, the expression levels of MRC1, a marker of alternatively activated macrophages (AAMs), did not change significantly in WT mice; however, its levels were markedly increased in IEX-1^−/−^ mice (lower panel of Fig. 4A), suggestive of increased presence of anti-inflammatory AAMs in eWAT of KO mice.

Analysis for hepatic inflammatory cytokines expression demonstrated that HFD feeding increased the expression of TNF-α and IL-1β in WT mice, an effect that was abrogated in IEX-1^−/−^ mice (top panel of Fig. 4B). Moreover, HFD-induced increases in the expression of hepatic adipogenic genes PPARγ and FABP4 were significantly inhibited in IEX-1^−/−^ mice (mid panels of Fig. 4B). Similar to the observation in eWAT, the expression levels of IL-10 were significantly higher in KO mice on HFD when compared to WT littermates (lower panel of Fig. 4B). This effect may have partially contributed to the improved hepatic steatosis (Fig. 3F) observed in KO mice^20^,^21^. Taken together, these data suggest that IEX-1^−/−^ mice are resistant to HFD-induced adipose and hepatic inflammation with increased expression of anti-inflammatory factors.

### IEX-1 deficiency increases energy expenditure as a result of enhanced thermogenesis

**Figure 5:**
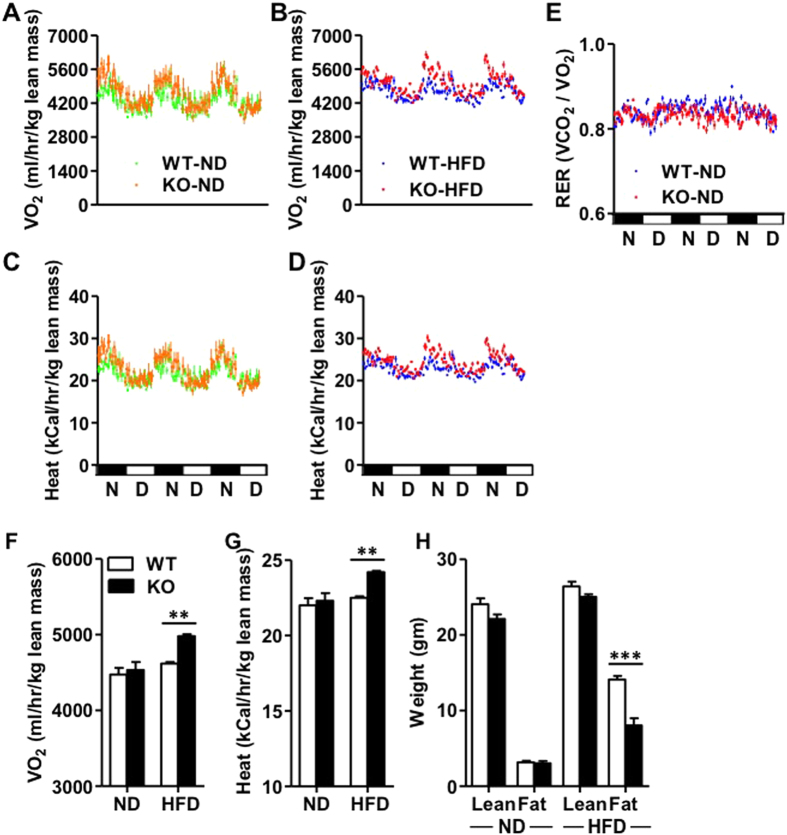
Increased energy expenditure in IEX-1 KO mice on high fat diet. Indirect calorimetry assessment of for 3 consecutive days showed no significant difference in (**A**) oxygen consumption (VO_2_) and (**C**) heat production between IEX-1 KO and WT mice when on ND. However, (**B**) VO_2_ and (**D**) heat production both significantly increased in KO mice when fed a HFD for 7–8 weeks as compared to WT littermates on same diet. (**E**) No difference in respiratory exchange ratio (RER) between two strains of mice was observed when on HFD. Mean analysis of 3 days measurements shown in (**A**–**D**) indicated significantly greater (**F**) VO_2_ and (**G**) heat production in IEX-1 KO mice (fill) than WT littermates (empty) on HFD. The values presented in (**A**–**G**) are normalized by lean body mass. (**H**) Analysis for lean and fat mass in mice fed with HFD or ND for 8 weeks revealed that IEX-1 deficiency resists the gain in fat mass after HFD without affecting lean mass. *n* = 4–5. **p < 0.01, ***p < 0.001 as indicated. N – night; D – Day.

The findings that IEX-1^−/−^ mice on HFD exhibited greater food intake with less weight gain suggested that KO mice may have increased energy expenditure. To investigate this, we measured energy consumption for 3 consecutive days in mice fed with ND or HFD for 7–8 weeks using indirect calorimetry. This analysis revealed that oxygen consumption (VO_2,_ Fig. 5A) and heat production (Fig. 5C) were comparable between IEX-1^−/−^ and WT mice when fed a ND. However, both VO_2_ (Fig. 5B) and heat production (Fig. 5D) increased in KO mice as compared to WT control when fed a HFD. The respiratory exchange ratio (RER = VCO_2_/VO_2_) fluctuated between 0.8 and 0.9 and was comparable between two strains of mice on HFD (Fig. 5E), suggesting that increased energy expenditure in IEX-1 deficiency is not due to change in fuel selection. The mean value analysis of 3 consecutive day’s measurements indicated significantly greater VO_2_ (4981 ± 26 versus 4619 ± 19 ml/hour/kg lean mass, Fig. 5F) and heat production (24 ± 0.1 versus 22 ± 0.1 kcal/hour/kg lean mass, Fig. 5G) in KO mice than WT control on HFD, suggesting elevated energy expenditure and thermogenesis in the absence of IEX-1. Furthermore, Echo-MRI of mice at 8 weeks of ND or HFD feeding demonstrated that lean phenotype of IEX-1^−/−^ mice was due to less gain in fat mass without affecting lean mass, providing another evidence for enhanced thermogenesis in these mice (Fig. 5H). These findings suggest that IEX-1 deficiency increases energy expenditure by elevating thermogenesis during high fat intake.

**Figure 6:**
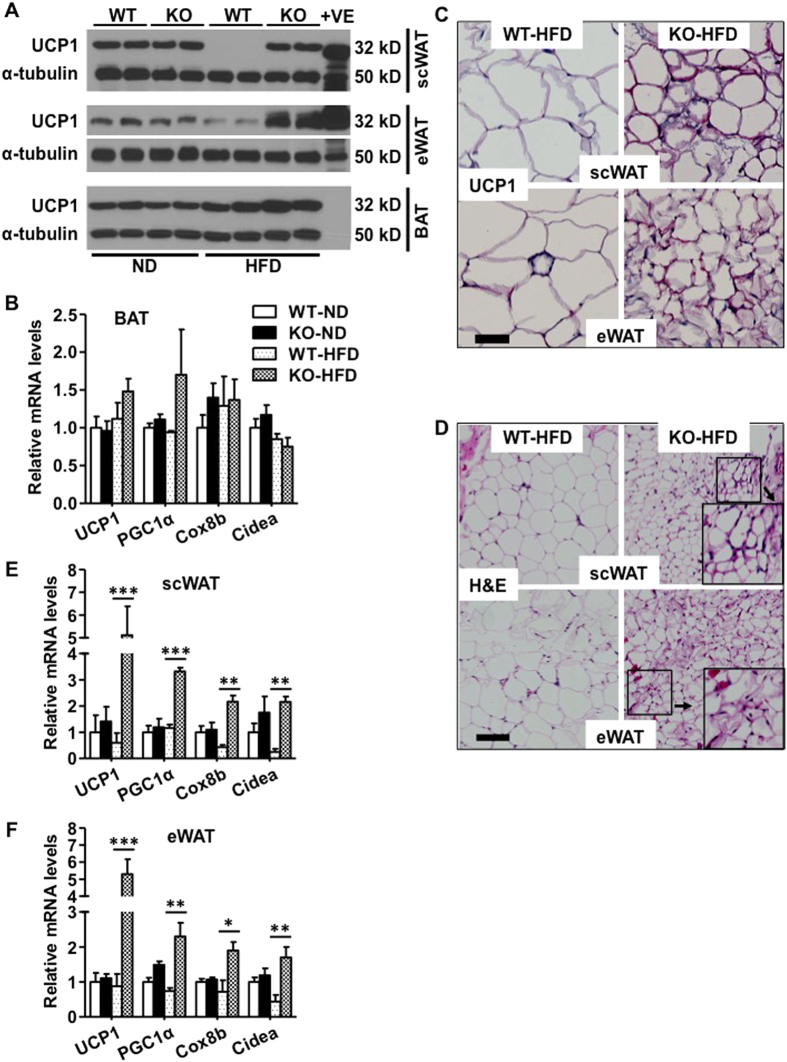
IEX-1 deficiency induces browning of white adipose tissues. (**A**) Immunoblotting of the whole cell lysate of indicated tissues obtained from mice fed with HFD or ND for 8 weeks demonstrated no difference in UCP1 protein levels in scWAT, eWAT, and BAT between two IEX-1 KO and WT mice when on ND. HFD feeding markedly suppressed UCP1 levels in scWAT and eWAT but not in BAT in WT mice. This effect was completely inhibited in KO mice, giving rise to greater levels of UCP1 in KO than in WT mice. α-tubulin was used as a loading control for UCP1. Each lane represents sample from individual animal (**B**) qRT-PCR analysis demonstrated no major impact of IEX-1 deficiency on thermogenic genes expression in BAT of mice on either diet described under (**A**). (**C**) Immunohistochemistry staining of scWAT (upper panel) and eWAT (lower panels) sections of these mice further showed increased UCP1 activity (purple) in KO mice on HFD as compared to WT control. (**D**) Hematoxylin and eosin (H&E) staining of the scWAT (upper panels) and eWAT (lower panels) sections displayed the appearance of multilocular adipocytes (insets) in KO but not in WT mice on HFD. qRT-PCR analysis revealed increased expression of thermogenic genes as indicated in both scWAT (**E**) and eWAT (**F**) of IEX-1 KO mice on HFD as compared to WT littermates (*n* = 4–6). Shown in (**A**,**C** and **D**) are representative immunoblots and images of 3 independent experiments. *p < 0.05, **p < 0.01, ***p < 0.001 as indicated. Bar in C and D = 50 and 100 μM, respectively.

Increased energy expenditure in IEX-1^−/−^ mice with no change in fuel preference prompted us to investigate whether elevated thermogenesis may have driven the lean phenotype of these mice. Brown fat is the primary site for non-shivering thermogenesis; therefore, we analyzed the expression of thermogenic markers in BAT by using Immunoblotting and qRT-PCR. We failed to detect any significant difference in UCP1 protein expression, the primary thermogenic molecule, between IEX-1^−/−^ and WT mice on either diet (Fig. 6A). Similarly, expression levels of BAT thermogenic genes *UCP1*, *PGC1α*, *Cox8b*, and *Cidea* were also comparable between two strains of mice (Fig. 6B), suggesting no major impact of IEX-1 deficiency on BAT function. Because HFD increased IEX-1 expression in eWAT and scWAT, we next analyzed these tissues for thermogenesis since these two depots of WAT are highly responsive to the thermogenesis inducers such as cold or β3 adrenergic agonist^22^,^23^,^24^,^25^. The UCP1 protein levels in scWAT and eWAT were comparable between IEX-1^−/−^ and WT mice on ND. Interestingly, HFD feeding for 8 weeks markedly reduced its levels in both scWAT and eWAT of WT mice (Fig. 6A). This effect was however completely inhibited in IEX-1^−/−^mice, giving rise to elevated UCP1 levels in both scWAT and eWAT of KO mice on HFD (Fig. 6A). Immuno-histochemistry and histologic analysis further showed an increased UCP1 signal (Fig. 6C) and recruitment of multilocular adipocytes (Fig. 6D) in both scWAT and eWAT of KO mice on HFD as compared to their WT counterparts, indicating browning/beiging of these tissues. Accordingly, expression of thermogenic genes *UCP1*, *PGC1α*, *Cox8b*, and *Cidea* was highly induced in scWAT (Fig. 6E) and eWAT (Fig. 6F) in IEX-1^−/−^ mice on HFD as compared to their WT control. No major difference in these parameters was observed between two strains of mice when fed with ND. Collectively, these data suggest that IEX-1 deficiency increases thermogenesis by activating thermogenic genes and inducing browning of WAT. These findings reveal a previously unknown role of IEX-1 in regulation of beige fat formation and energy metabolism.

### IEX-1 deficiency prevents HFD-induced switch in adipose tissue macrophage phenotype

**Figure 7:**
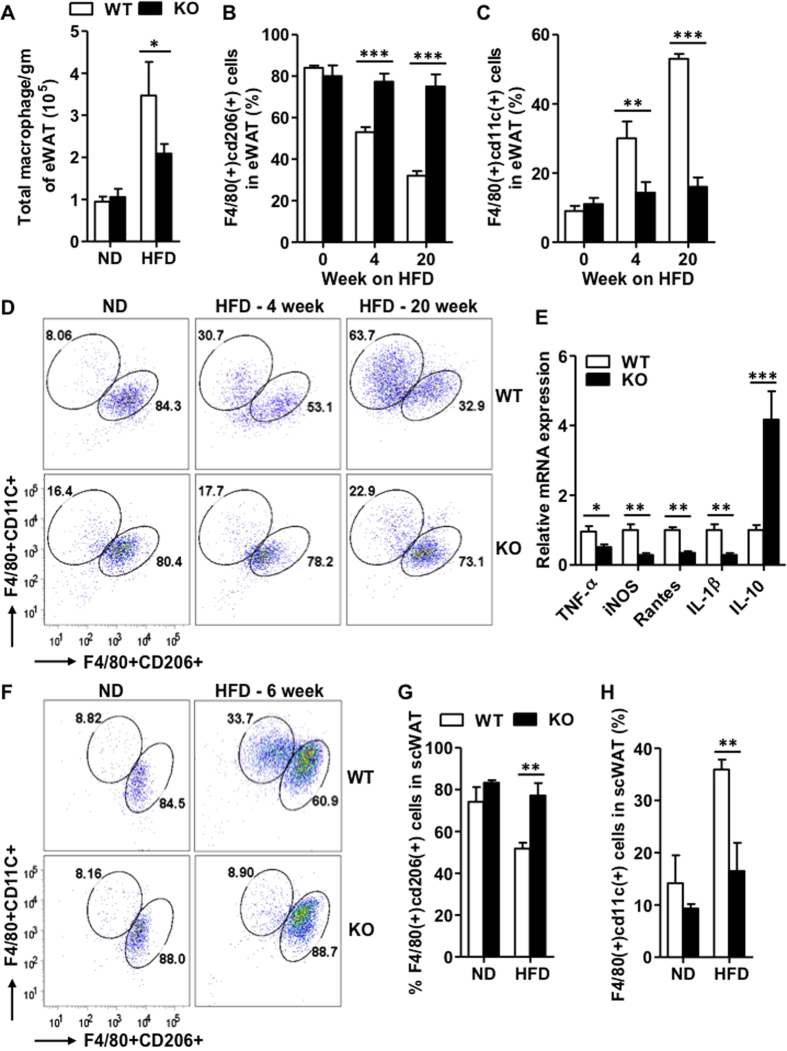
IEX-1 deficiency inhibits HFD-induced switch in adipose tissue macrophage phenotype. (**A**) HFD feeding for 20 weeks increased the number of total macrophages in eWAT of WT mice (empty), an effect that was partially inhibited in KO mice (fill) as determined by FACS analysis (*n* = 5–7). Phenotypic analysis of eWAT macrophages revealed that HFD strongly reduced (**B**) the percentage of AAMs (F4/80 + cd206+) while increasing (**C**) the percentage of CAMs (F4/80 + cd11c+) in WT mice both after 4 and 20 week (*n* = 5–7). However, these effects were not seen in IEX-1 KO mice. (**D**) The representative FACS analysis for eWAT macrophage phenotype of 5–7 independent experiments with similar results is shown, indicating that KO mice sustained majority of adipose AAMs. (**E**) qRT-PCR analysis demonstrated diminished expression of pro-inflammatory cytokines TNF-α, iNOS, Rantes, and IL-1β whereas increased levels of anti-inflammatory cytokine IL-10 in adipose macrophages from IEX-1 KO mice fed with HFD for 20 weeks as compared to WT counterparts (*n* = 4). (**F**) The representative FACS analysis of macrophages obtained from scWAT of mice fed with HFD or ND for 6 weeks, demonstrating that HFD reduced the percentage of AAMs and increased the percentage of CAMs in WT mice (upper panels). This effect was abrogated in scWAT of IEX-1 KO mice (lower panels). Quantitative analysis of FACS charts shown in (**F**) for percentages of AAMs (**G**) and CAMs (**H**) in scWAT (*n* = 3). *p < 0.05, **p < 0.01, ***p < 0.001 as indicated.

The finding that IEX-1^−/−^ mice on HFD showed marked increases in MRC1 and IL-10 expression in eWAT, suggests the enhanced availability of AAMs in adipose tissue because AAMs strongly express these markers^18^,^26^. Adipose AAMs play a crucial role in the biogenesis of beige fat by secreting catecholeamines^22^,^23^,^24^, therefore, we determined adipose tissue macrophages (ATMs) phenotype in stromal vascular fraction (SVF) of eWAT of mice fed with HFD or ND for 20 weeks^18^. There was no difference in the number of ATMs and the percentages of AAMs (F4/80 + cd206 + cd11c-) and CAMs (F4/80 + cd11c + cd206-) in eWAT between IEX-1^−/−^ and WT mice on ND (Fig. 7A–D). The number of ATMs increased by ~3.5-folds after 20 weeks of HFD in WT mice, an effect that was partially inhibited in KO mice (2 ± 0.2 versus 3.5 ± 0.8 × 10^5^ macrophages/gm of WAT, Fig. 7A). The majority of ATMs in eWAT existed in AAM-polarized state in both WT and KO mice when fed ND. However, the percentage of AAMs sharply decreased from 84 ± 1% to 30 ± 2% (Fig. 7B and right panels D) concomitant with an increase of CAMs from 9 ± 1% to 53 ± 1% (Fig. 7C and right panels D) in WT mice fed with HFD. This observation is in consistence with the previous reports demonstrating a phenotypic switch of ATMs from AAM to CAM in response to HFD^18^,^27^. In sharp contrast, the percentages of AAMs and CAMs remained unaltered in IEX-1^−/−^ mice even after 20 weeks of HFD feeding (Fig. 7B,C and lower panels D). Thus, IEX-1^−/−^ mice sustained majority of their adipose AAMs population.

Since KO mice gained less weight on HFD than did their WT littermates, the disparity in ATM phenotype might be a secondary consequence of marked difference in their body weight after 20 weeks of HFD. To address this possibility, we analyzed ATMs phenotype in mice fed with HFD for only 4 weeks when there was no difference in their body weight (Fig. 2A). Notably, even 4 weeks of HFD feeding induced a significant transition in ATMs phenotype in WT mice, decreasing AAMs percentage from 84 ± 1% to 53 ± 2% while increasing CAMs from 9 ± 1% to 30 ± 5%, and (Fig. 7B,C and mid panels D). Once again, the percentage of these cells remained unaltered in IEX-1^−/−^ mice that sustained basal level of AAMs. Moreover, to determine whether differential ATM phenotype is also reflected in the inflammatory status of these cells, we isolated ATMs from eWAT of mice fed with HFD. The qRT-PCR analysis demonstrated the diminished expression of pro-inflammatory cytokines TNF-α, iNOS, rantes, and IL-1β in ATMs lacking IEX-1 as compared to their WT counterparts (Fig. 7E). In contrast the expression of anti-inflammatory cytokine IL-10 was markedly greater in the absence than in the presence of IEX-1 (Fig. 7E). This data further corroborates the differential ATM phenotype observed in two strains of mice on HFD. Since, browning was also prominent in scWAT in IEX-1 deficiency; we determined ATMs phenotype in SVF of scWAT of mice fed with HFD or ND for 6 weeks. Similar to our observation in eWAT, HFD feeding in WT mice decreased the percentage of AAMs from 75 ± 7% to 51 ± 3%, while increasing CAMs from 14 ± 5% to 35 ± 2% (Fig. 7F–H), demonstrating the HFD-induced transition in ATMs phenotype in scWAT. However, this switch in ATMs phenotype was not observed in IEX-1 KO mice. No difference in ATM phenotype was observed when mice were fed with ND. Collectively, these data suggest that HFD-induced AAMs to CAMs transition in adipose tissue occurs well before the inception of obesity and that this phenomenon requires IEX-1. This data further indicates that IEX-1 deficiency induces browning of WAT by promoting alternative activation of ATMs.

## Discussion

Obesity is a chronic disorder that occurs as a result of imbalance in energy metabolism^2^,^3^,^5^,^6^,^8^. The primary physiological regulators of energy expenditure especially during high calorie intake are not fully understood. Here we report a previously unknown role for IEX-1 in energy metabolism. Null mutation of IEX-1 protects mice from HFD-induced obesity development and insulin resistance. IEX-1^−/−^ mice exhibited increased energy expenditure due to enhanced thermogenesis. The tissue-specific investigations reveal that IEX-1 deficiency increases thermogenic genes expression and induces browning of eWAT and scWAT, which is in consistence with the observation of increased IEX-1 expression exclusively in these tissues on HFD feeding. This explains the elevated thermogenesis and energy expenditure in KO mice, providing a mechanistic insight into the lean phenotype of these mice. The study identifies IEX-1 as an important modulator of energy metabolism during high calorie intake and offers a novel target for treating obesity and other metabolic disorders.

We asked where the primary effect of IEX-1 was during HFD consumption and how IEX-1 deficiency increased energy expenditure and protected mice against HFD-induced obesity. IEX-1 is a direct transcriptional target of NF-кB that plays a crucial role in the pathogenesis of obesity^5^. Similar to NF-кB, IEX-1 expression is highly induced in WAT and in the infiltrated immune cells in mice upon HFD feeding. In consistence with this, IEX-1 deficiency induces beige fat formation in scWAT and eWAT without affecting BAT function during HFD feeding as evident by activation of thermogenic genes and recruitment of UCP1 + adipocytes. Accordingly, IEX-1^−/−^ mice also displayed increased body temperature, thermogenesis and elevated energy expenditure with no change in fuel selection. Beige adipocytes are brown fat-like cells that are recruited in WAT and express high levels of UCP1. Induction of UCP1 in WAT and converting white adipocytes into brown (browning) can efficiently increase energy expenditure by elevating thermogenesis. Thus, recruitment of beige adipocytes by several pharmacological means effectively inhibits obesity development by increasing energy expenditure^23^,^24^,^28^,^29^,^30^. The increased energy expenditure in IEX-1^−/−^ mice primarily reflects the thermogenic activity of beige fat in scWAT and eWAT because interscapular BAT UCP1 content, RER, and muscle UCP1 gene expression (data not shown) were not different among the genotypes. Thus, recruitment of beige fat in WAT is most likely the primary mechanism by which IEX-1 deficiency inhibits HFD-induced obesity. However, this study does not rule out a role of IEX-1 in other metabolic organs/tissues or cell types that may have contributed to the lean phenotype observed in IEX-1 KO mice. IEX-1 has been reported to express in pancreas (islet cells), small intestine (epithelium), and in thyroid follicles (epithelial cells)^31^. Whether IEX-1 activity in WAT represents its major metabolic action or other tissues are also involved warrants further studies.

IEX-1 deficiency induced browning of adipose tissue probably by increasing AAMs in WAT. Several recent landmark studies have provided strong evidence suggesting that ATMs are a crucial component of the efferent pathway required for biogenesis of functional beige fat. Beiging stimuli such as cold triggers recruitment of AAMs in adipose tissue^22^,^23^,^24^,^29^,^32^. These adipose AAMs then secret catecholeamines that act on the surrounding adipocytes precursors to induce beige fat formation and activation of thermogenic genes^22^,^23^,^24^. Conversely, loss of AAM polarization or deletion of macrophages prevents cold-induced beige fat formation, underscoring a crucial role of AAMs in beige fat biogenesis^22^,^23^. The majority of ATMs in lean animals exist in AAM polarized state to maintain tissue homeostasis and inhibit inflammation. HFD induces a switch in ATMs phenotype from AAM in lean animals to CAM in obese fat^18^,^27^. Likewise, we also observed an AAM to CAM transition in ATMs phenotype in WT mice after HFD feeding. In sharp contrast, IEX-1^−/−^ mice did not show such transition even after 20 weeks of HFD feeding and sustained more than 90% of adipose AAMs. These AAMs express low levels of inflammatory cytokines, a hallmark of AAMs^18^. Because, AAMs stimulate beiging by releasing catecholeamines, the sharp increase in the availability of adipose AAMs in KO mice could induce the browning of WAT in these animals. The reduction in WAT UCP1 expression concomitant with a sharp decline in AAM population in WT mice after HFD feeding further supports this notion. The strikingly different ATMs phenotype in KO mice may not be a consequence but rather a cause of browning induced-reduced weight gain in these animals because the differential ATMs phenotype was also evident at 4 weeks of HFD, when body weight was same between two strains of mice. These data suggest that IEX-1 may be an important negative, physiological regulator of beige fat biogenesis likely by inhibiting AAM polarization.

How IEX-1 deficiency sustains adipose AAMs remains to be elucidated. IEX-1 is highly expressed in macrophages^11^,^12^,^14^. It plays a crucial role in regulation of apoptosis in immune cells and controls their heterogeneity^9^,^12^,^16^,^33^,^34^,^35^,^36^. For example we have previously reported that IEX-1 reciprocally regulates the T-cell survival and apoptosis in a subset-dependent fashion^16^,^36^. IEX-1 deficiency increases apoptosis of Th1 while promoting the survival of Th17 cells, leading to enhanced IL-17 response in mouse models of colitis and arthritis. Perhaps, HFD-induced IEX-1 activity in macrophages preferentially increases the survival of CAM subset but not AAMs in obese fat by its putative antiapoptotic action. Therefore, IEX-1 deficiency prevents HFD-induced increase in CAMs population. Nevertheless, our data suggest that IEX-1 is required for HFD-induced switch in ATM phenotype thereby regulating AAMs-mediated beiging.

Adipose macrophages play a crucial role in obesity-associated inflammation by switching their phenotype from an anti-inflammatory state (AAM) to pro-inflammatory state (CAM) in obese fat^7^,^18^,^37^,^38^. IEX-1 deficiency inhibited such ATMs transition and thus AAMs were predominant ATMs in WAT of IEX-1^−/−^ mice even after 20 weeks of HFD, providing a mechanism by which IEX-1 deficiency inhibited HFD-induced inflammation. Presumably, increased AAMs population and attenuated inflammation in IEX-1^−/−^ mice may have contributed to the improved insulin sensitivity observed in these mice^32^,^39^. IEX-1 may represent a novel mediator of obesity-associated inflammation likely by its role in regulation of ATMs phenotype. Thus, we propose that IEX-1exerts a dual action in obesity via ATMs; (i) it promotes CAM-induced inflammation and (ii) inhibits beige fat biogenesis.

In conclusion, the present study identified a previously unappreciated role for IEX-1 in energy regulation. IEX-1 deficiency induced browning and activated thermogenic genes in WAT by promoting alternative activation of adipose macrophages during HFD feeding. Consequently, IEX-1^−/−^ mice were protected from HFD-induced weight gain due to enhanced thermogenesis and increased energy expenditure. Alternative activation of macrophages also attenuated HFD-induced inflammation and improved insulin resistance. Whether sustenance of AAMs is the primary mechanism by which IEX-1 deficiency inhibits obesity development however remains to be determined.

## Methods

### Animals and animal care

All mouse studies were conducted in accordance with the NIH Guide for the Care and Use of Laboratory Animals. All studies were reviewed and approved by and in compliance with the MGH Subcommittee on Research Animal Care. IEX-1^−/−^ mice were generated in our lab as described previously^17^. Male mice on 129Sv/C57BL/6 background were used in this study. IEX-1^−/−^ mice and their WT littermate fed a high fat diet (45% calories from fat; D12451 Research Diets Inc) or normal chow (13.2% calories from fat) starting at 8 weeks of age for 20 weeks. Animals were housed in a specific pathogen-free facility with a 12-hour light/12-hour dark cycle in the animal facilities of MGH and given food and water *ad libitum*. Rectal temperature recordings were determined with FHC Precision thermometer (FHC Bowdoin ME) around noon. Body weight was monitored every 2 week throughout the study.

### Whole blood and plasma measurements

Whole blood was collected into heparinized tubes and plasma was separated by centrifugation. Plasma insulin concentrations were measured by insulin ELISA kit (Crystal Chem Inc, Downers Grove IL). Blood glucose was measured by One Touch Ultra Accuchek Glucometer. Plasma total cholesterol, triglycerides and NEFA were measured by colorimetric assay (Wako, Cambridge MA). Plasma TNF-α and IL-10 were measured by ELISA kits (eBioscience).

### Glucose and insulin tolerance tests

For IGTT, mice were injected intraperitoneally with 1.5 mg glucose/g body weight after 12 hrs of fasting^5^. Blood glucose was measured at basal, 15, 30, 45, 60, and 120 min from tail blood using the glucometer. For ITT, mice were given an intraperitoneal injection of 0.75 unit human insulin (Novolin, Novo Nordisk) per kg body weight after 3 hrs of fasting^5^. Blood glucose concentrations were determined as described above.

### Histology

After dissection, specimens were fixed by immersion in 10% buffered formalin, for 48–72 hours, dehydrated, cleared, and then embedded in paraffin. Serial sections (5 μm thick) were obtained and deparaffinized, rehydrated and then stained by hematoxylin and eosin. The sections images were then scanned using a NanoZoomer and analyzed using NDP view software (Hamamatsu, Bridgewater, NJ). Mean adipocyte area of 100 randomly sorted adipocytes per specimen of eWAT sections was determined using NDP view software.

### Immunohistochemistry

Formalin-fixed paraffin-embedded tissue sections were deparaffinized and rehydrated prior to antigen unmasking by boiling in 10 mM sodium citrate and permeabilization with 0.1% TBS-Triton for 15 min. Endogenous peroxidase activity was quenched with incubation with 3% hydrogen peroxide for 15 minutes. Sections were blocked in normal goat serum and incubated with primary rabbit anti-IEX-1 (ab65152 Abcam, Cambridge MA) or anti-UCP1 (ab10983 Abcam) antibody overnight at 4 ^o^C. Sections were then stained with biotinylated secondary antirabbit antibody for 1 hour at room temperature and color was visualized with 3,3′-diaminobenzidine (DAB) using the Vecta -stain ABC kit (Vector Laboratories). Sections for UCP1 detection were stained with polymer AP anti-rabbit secondary antibody for 30 min at room temperature (Biocare Medical, Brookline MA) and color was visualized by adding fast red chromogen (Biocare Medical). Sections were counterstained with hematoxylin prior to dehydration and coverslip placement with mounting media. The slides were scanned using a NanoZoomer and analyzed using NDP view software (Hamamatsu, Bridgewater, NJ).

### Immunoblotting

Tissues were harvested in RIPA phosphate buffer supplemented with protease and phosphatase inhibitors (Sigma-Aldrich). Extracts were fractionated using 4–20% Miniprotein TGX gel (BioRad, Waltham MA), transferred to nitrocellulose membranes, and incubated with primary antibodies against mouse IEX-1 (1:200; sc33171 Santa Cruz Biotech, Dallas TX), UCP1 (1:5000 of eWAT and scWAT and 1:20,000 for BAT, ab10983 Abcam), or α-tubulin (abcam) overnight at 4 ^o^C. After washing membranes were incubated with HRP-linked anti–rabbit IgG (Cell Signaling Technology). Membranes were incubated with ECLPlus (GE Healthcare, Wilmington, MA), and chemifluorescence was detected with a Versadoc 4000MP imager. Captured images were analyzed with ImageJ software (NIH).

### SVF isolation and immunophenotyping

4–5 male mice per genotype were euthanized under deep anesthesia with a mixture of Ketamine (120 mg/kg) + Xylazine (12 mg/kg) administered intraperitoneally. Epididymal WAT were collected and minced with scissors and digested with 1 mg/ml of collagenase II in PBS containing CaCl_2_ (1.4 mM) and BSA (0.5%) for 30 minutes at 37 °C in a shaker. The digestion was stopped by adding equal amount of DMEM containing 10% FBS. The cell suspension was filtered through a 100 μm filter and then spun at 300*g* for 5 minutes to separate floating adipocytes from stromal vascular fraction (SVF) pellet. SVF pellet was resuspended in 0.5 ml RBC lysis buffer for 5 min on ice, centrifuged for 5 min at 300 g and resuspended in FACS buffer (PBS with 2% BSA) at a concentration of 6 × 10^6^ cells/ml. Cells were incubated in dark on ice for 20 minutes in Fc block (BD Pharmingen, San Jose, CA). Without washing cells were incubated with fluorescently labeled antibodies: F4/80-phycoerythrin, CD11c-phycoerythrin-Cy7 and CD206-Alexaflour 647 for additional 30 minutes as instructed by manufacturer (BD Bioscience). The cells were analyzed using FACS Aria cell sorter (BD Biosciences). Unstained and single stains were used for setting compensations and gates.

### RNA extraction and quantitative real-time PCR analysis

The tissues or adipocytes and SVF fractions of eWAT were obtained from mice under deep anesthesia as described above and stored at −80 ^o^C until extraction. Total RNA was extracted using Trizol (Invitrogen, Carlsbad CA). cDNA was synthesized using Superscript III first strand synthesis kit (Invitrogen). The mRNA expression levels were measured by quantitative real-time PCR analysis (qRT-PCR) in a Mastercycler realplex 2 (Eppendorf) using Kapa sybr fast or probe fast qPCR mix (Kapa Biosystems) using conventional Sybr or taqman (For IEX-1) primers. Changes in relative gene expression were normalized to 18S mRNA levels and were determined using the relative Ct method.

### Whole animal energy expenditure and heat production

IEX-1^−/−^ mice and WT littermates (*n* = 4–5 per genotype) fed with either HFD or ND for 7–8 weeks were placed in standard metabolic cages. The oxygen consumption (VO_2_), carbon dioxide production (VCO_2_), heat production, spontaneous motor activity and food intake were measured using the Comprehensive Laboratory Monitoring System (TSE system Inc), an integrated open-circuit calorimeter equipped with an optical beam activity monitoring system. These parameters were monitored during 3 consecutive days (3 dark cycles and 3 light cycles) and the mean values for 3 days were used in the analyses. Respiratory exchange ratio (RER) was calculated as a ratio of VCO_2_ to VO_2_. Body composition was measured by Echo MRI analyzer (Medical Device Company, Houston TX).

### Statistical analysis

All data are expressed as mean ± standard errors of mean (SEM). Differences between two groups or two treatments including IEX-1 mRNA and protein levels in ND and HFD fed WT mice, and cytokines mRNA expression in ATMs were compared with a Student’s two-tailed *t* test. Two-Way repeated measures ANOVA was used to compare body weight, blood parameters, blood glucose during IGTT and ITT, mRNA expression levels in eWAT, scWAT, BAT and liver, VO_2_ and heat production, and percentages of CAMs and AAMs in SVF among different groups. P values < 0.05 were considered statistically significant.

## Additional Information

**How to cite this article**: Shahid, M. *et al*. IEX-1 deficiency induces browning of white adipose tissue and resists diet-induced obesity. *Sci. Rep*. **6**, 24135; doi: 10.1038/srep24135 (2016).
